# The contribution of the rs55705857 G allele to familial cancer risk as estimated in the Utah population database

**DOI:** 10.1186/s12885-019-5381-2

**Published:** 2019-03-01

**Authors:** Sarah Hummel, Wendy Kohlmann, Thomas M. Kollmeyer, Robert Jenkins, Joshua Sonnen, Cheryl A. Palmer, Howard Colman, Diana Abbott, Lisa Cannon-Albright, Adam L. Cohen

**Affiliations:** 1 George E. Wahlen Department of Veterans Affairs Medical Center, Salt Lake City, Utah USA; 2 Division of Genetic Epidemiology, Department of Internal Medicine, University of Utah School of Medicine, Salt Lake City, Utah USA; 3 Division of Oncology, University of Utah School of Medicine, Huntsman Cancer Institute, Salt Lake City, Utah USA; 4 Department of Neurosurgery, University of Utah School of Medicine, Huntsman Cancer Institute, Salt Lake City, Utah USA; 5 Department of Human Genetics/Pediatric Division of Medical Genetics, Graduate Program in Genetic Counseling, University of Utah School of Medicine, 15 North 2030 East, Salt Lake City, 84112 Utah USA; 6 The Mayo Clinic, Department of Laboratory Medicine and Pathology, Rochester, Minnesota USA; 7 Department of Population Sciences, University of Utah School of Medicine, Huntsman Cancer Institute, Salt Lake City, Utah USA; 8 Division of Anatomic Pathology, University of Utah School of Medicine, Salt Lake City, Utah USA

**Keywords:** Molecular epidemiology, IDH, rs55705857, Oligodendroglioma, Cancer

## Abstract

**Background:**

*IDH1/2* mutated glioma has been associated with a germline risk variant, the rs55705857 G allele. The Utah Population Database (UPDB), a computerized genealogy of people in Utah, is a unique resource to evaluate cancer risk in related individuals.

**Methods:**

One hundred and two individuals with *IDH1/2* mutant or 1p/19q co-deleted glioma were genotyped and linked to the UPDB. DNA came from blood (21), tumor tissue (43), or both (38). We determined congruence between somatic and germline samples and estimated the relative risk for developing cancer to first and second-degree relatives of G and A allele carriers at rs55705857.

**Results:**

Somatic (glioma) DNA had 85.7% sensitivity (CI 57.2–98.2%) and 95.8% specificity (CI 78.9–99.89%) for germline rs55705857 G allele. Forty-one patients were linked to pedigrees in the UPDB with at least three generations of data. First-degree relatives of rs55705857 G allele carriers were at significantly increased risk for developing cancer (RR = 1.72, *p* = 0.045, CI 1.02–2.94), and specifically for oligodendroglioma (RR = 57.61, *p* = 0.017, CI 2.96–320.98) or prostate cancer (RR = 4.10, *p* = 0.008, CI 1.62–9.58); relatives of individuals without the G allele were not at increased risk. Second-degree relatives of G allele carriers also had significantly increased risk for developing cancer (RR = 1.50, *p* = 0.007, CI 1.15–2.01).

**Conclusions:**

Tumor DNA may approximate genotype at the rs55705857 locus. We confirmed this locus confers an increased risk of all cancers and especially of oligodendroglioma. No increased cancer or brain tumor risk is seen in family members of individuals without the high-risk G allele.

**Electronic supplementary material:**

The online version of this article (10.1186/s12885-019-5381-2) contains supplementary material, which is available to authorized users.

## Background

Germline genetic testing is a powerful tool that can yield important predictive information about a person’s future health [1–3]. Detection of germline pathogenic mutations can lead to improved screening, additional prevention strategies, and better understanding of personal cancer risk [1–3]. Gliomas are categorized by somatic genetic/molecular profiling in addition to histology in order to improve treatment targets or provide prognostic information [3–6]. Germline genetic associations with specific somatic molecular subtypes are just beginning to be understood [2–4, 6, 7].

Glioma is the most common type of primary brain cancer, with an overall glioma incidence of approximately 5 per 100,000 persons per year [8]. Gliomas are now characterized based on the presence of mutations in the isocitrate dehydrogenase family of genes (*IDH1* and *IDH2*) [6]. Mutations in *IDH1* or *IDH2* are found in 100% of oligodendrogliomas, 70–80% of lower grade astrocytomas, and in secondary glioblastoma [9]. Co-deletion of chromosomes 1p and 19q can be used as a surrogate for *IDH* mutation because 1p/19q co-deletion is invariably associated with *IDH* mutation [10].

Many studies have found genetic contributions to familial glioma risk (RR ~ 2.0–3.8), but the original studies were unable to identify the origin of the increased risk [11, 12]. Recently, genome wide association studies have consistently identified the rs55705857 G allele at 8q24 as a risk factor for gliomagenesis that is specific for *IDH1/2* mutated gliomas [5, 7, 13–18]. Approximately 40% of individuals with *IDH1/2* mutated oligodendrogliomas and astrocytomas carry the germline rs55705857 G allele, compared with approximately 8% of the general population [5, 13]. The identification of rs55705857 G allele as a contributing factor to glioma development specific for *IDH* mutated gliomas provides an opportunity to more precisely calculate risks to relatives of patients with glioma.

All prior studies of risk alleles have been case-control or GWAS studies. We explore the heritability of glioma associated with the rs55705857 G allele in a population based cohort database. We used the Utah Population Database (UPDB), a computerized resource that links data from the Utah Cancer Registry, Utah birth and death certificates, and Utah driver licenses, among other data sources. Over 6.5 million individuals have data linked to the UPDB computerized resource. Data available from the Utah Cancer registry, a Surveillance, Epidemiology and End Results registry, spans from 1973 to 2012 and includes primary site, histology, age at diagnosis, stage, grade, survival, and treatment data. Over 2.5 million individuals have at least three generations of genealogy data that connects to the original Utah genealogy [19]. The UPDB resource allowed us to evaluate the association between the rs55705857 G allele and several types of *IDH* associated cancers in relatives of individuals with *IDH1/2* mutated glioma.

We hypothesized that first and second-degree relatives of patients with an *IDH1/2* mutated glioma and a germline rs55705857 G allele had higher risk for developing one or more of the following cancers in which *IDH1/2* mutations have been found: glioma, prostate, colon, hepatic, lymphomas, biliary tumors, primary myelofibrosis, central chondrosarcoma, chrondroma/enchondroma, thyroid, acute lymphoblastic leukemia, and acute myelogenous leukemia [20–22]. We also aimed to determine the accuracy of tumor tissue for assessing the rs55705857 G allele and the relative risk of specific cancers in first and second-degree relatives of individuals with *IDH1/2* mutated gliomas with and without the rs55705857 G allele.

## Methods

### Patient identification/Proband sample population

The population of this study was derived from the Huntsman Cancer Institute Cancer Clinical Research Database (CCR). Secondary analyses of patient data and specimens with a waiver of informed consent were approved by the University of Utah IRB and all research was conducted following the international standards set forth in the Declaration of Helsinki. Patients were identified using CPT and ICD-9 codes for histology and location to find Grade II-III oligodendroglioma, oligoastrocytoma, glioma NOS, and/or astrocytoma patients, and only individuals with tumors with documented *IDH1* mutations, *IDH2* mutations, or 1p/19q codeletion were included. Stored germline tissues, somatic tissues, or DNA was obtained from the HCI Biorepository. In total, 102 unique patients had DNA or tissue samples available and were genotyped for the G allele of rs55705857 SNP (Table 1).

**Table 1:** DNA sample characteristics of glioma patients harboring *IDH1/2* mutant or 1p/19q co-deletion

DNA Source	Male Patients	Female Patients
Somatic	15	28
Germline	10	11
Somatic and Germline	21	17

Somatic DNA was derived from tumor samples. Germline DNA was derived from blood samples. One hundred and two individuals had one or more sources of DNA available for analysis

### Genotyping

G allele genotyping at rs55705857 was performed by the Mayo Clinic Genotyping Core, utilizing a TaqMan Assay from ABI with Genotyping Master Mix. Amplification and post amplification genotype readings were performed on an Applied Biosystems HT7900. Samples were submitted in 96-well plates. Fifteen water blanks and replicate samples were plated at random.

We considered the patient to be germline positive for the rs55705857 G allele when at least one replicate of the sample had complete agreement with two runs detecting heterozygote/homozygote status for the G allele. Somatic DNA was considered positive for detection of the rs55705857 G allele when one of the two runs detected the G allele (Additional file 1: Table S1).

### Linking records to the UPDB

The Utah population is predominantly of Northern European ancestry with average rates of consanguinity similar to those for the United States and negligible genetic drift [23, 24]. We linked patients meeting our study criteria to the UPDB; after linkage, no identifiers were used. Record linkage and analyses were approved by The University of Utah Institutional Review Board and the Resource for Genetic Epidemiologic Research. There was no contact with human subjects. Familial cancer risk analyses were only performed on patients with at least three generations of genealogy.

### Analysis

Methods used to analyze genealogical data within the UPDB have been previously described in detail [11, 19, 25]. Our work estimated the Relative Risk (RR) of cancer in first and second-degree relatives of genotyped patients. The RR of cancer in relatives of genotyped patients is defined as the ratio of the observed number of cancers for a given set of relatives to the expected number of cancers. Expected numbers of cancers are based on cohort-specific population rates for each cancer, calculated from within the UPDB. Cohorts represent sex, birth state (Utah or not), and 5-year birth-year groups. The expected number of relatives with cancer was estimated by counting all relatives of the genotyped patients by cohort, then multiplying the number of relatives in a given cohort by the cohort-specific rate of each tumor subtype. That value was summed over all cohorts to create estimates of the RR for each cancer. Given a null hypothesis RR≤ 1, we calculated one-sided probabilities that RR > 1. We assumed the number of observed cancers followed a Poisson random variable with a mean equal to the expected number of cases.

## Results

**Fig. 1:**
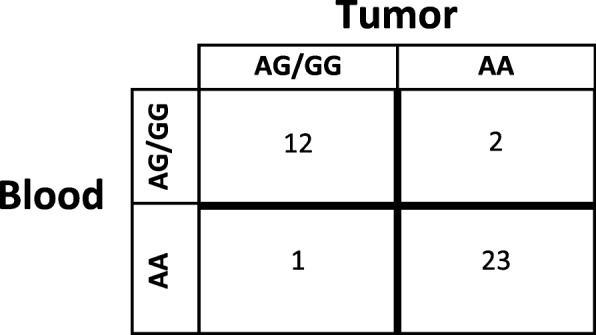
Accuracy of genotype in 38 individuals with somatic and germline DNA samples. Abbreviations: AA, rs55705857 A allele homozygote; AG, rs55705857 G allele heterozygote; GG, rs55705857 G allele homozygote. Calculations assume that DNA derived from blood is the true germline representation of the individual

Presence or absence of the rs55705857 G allele was successfully determined for one hundred two individuals (Table 1). We determined congruence between somatic and germline DNA for 38 of 102 (37%) individuals. We assumed DNA derived from blood or other non-tumor tissue was an accurate representation of germline DNA complement. Somatic (glioma) DNA had 85.7% Sensitivity (CI 57.2–98.2%) and 95.8% Specificity (CI 78.9–99.89%) for predicting the presence of the rs55705857 G allele in the germline. Somatic DNA had a positive predictive value (PPV) of 93.2% (CI 90.1–94.0%) and 90.9% negative predictive value (CI 89.2–91.3%). The overall accuracy of tumor genotype was 92.1% (CI 78.6–98.3%) (Fig. 1). Germline DNA was not available for all participants. Based on the high rate of concordance between the germline and somatic rs55705857 genotype, germline genotypes were inferred for those without germline DNA based on the somatic genotype.

Of the 102 genotyped individuals, forty-one individuals were linked to ≥3 generation UPDB genealogies. Eighteen of 41 subjects were found to have the rs55705857 G allele (G allele carriers). The G allele carriers had 140 first-degree relatives in UPDB; 13 cancers were observed in that population. The G allele carriers had 412 s-degree relatives with 44 cancers. Relative risk estimates for a subset of cancers are summarized in Table 2.

**Table 2:** Cancer incidence for 1st degree relatives of G allele carriers

Cancer Type	Obs	Exp	1 T *P*-Value	RR	95% CI
ANY CANCER	13	7.56	0.045	1.72	1.02–2.94
BRAIN	≤5	*	0.163	5.61	0.29–31.24
- OLIGODENDROGLIOMA	≤5	*	0.017	57.61	2.96–320.98
COLORECTAL	≤5	*	0.546	0	0–4.95
THYROID	≤5	*	0.750	0	0–10.43
PROSTATE	≤5	*	0.008	4.1	1.62–9.58

*Abbreviations:* *Obs* observed number of individuals with given cancer, *Exp* expected number of individuals with given cancer, *1 T P-Value* one-tailed *p*-value, *RR* relative risk, *CI* confidence interval

For all cancer sites with ≤5 observed cases, exact values are not shown for observed and expected to preserve patient anonymity per the Utah Resource for Genetic and Epidemiologic Research (RGE) requirements and are marked with an asterisk. Cancer incidence calculated in one hundred forty first-degree relatives

First-degree relatives of G allele carriers were at significantly increased risks for developing any cancer (RR = 1.72, *p* = 0.045, CI 1.02–2.94) and specifically for developing oligodendroglioma (RR = 57.61, *p* = 0.017, CI 2.96–320.98) or prostate cancer (RR = 4.10, *p* = 0.008, CI 1.62–9.58). Conversely, first-degree relatives of individuals who did not carry the high-risk G allele (rs55705857 A allele homozygotes) were not at significantly increased risk for developing cancer overall, or for developing any individual cancer tested (Table 3).

**Table 3:** Cancer incidence for 1st degree relatives of rs55705857 A allele homozygote individuals

Cancer Type	Obs	Exp	1 T P-Value	RR	95% CI
ANY CANCER	12	11.91	0.528	1.01	0.58–1.76
BRAIN	≤5	*	0.772	0	0–11.58
- OLIGODENDROGLIOMA	≤5	*	0.976	0	0–124.9
COLORECTAL	≤5	*	0.260	2.02	0.36–7.3
THYROID	≤5	*	0.665	0	0–7.35
PROSTATE	≤5	*	0.097	0	0–1.28

*Abbreviations:* *Obs* observed number of individuals with given cancer, *Exp* expected number of individuals with given cancer, *1 T P-Value* one-tailed *p*-value, *RR* relative risk, *CI* confidence interval

For all cancer sites with ≤5 observed cases, exact values are not shown for observed and expected to preserve patient anonymity per the Utah Resource for Genetic and Epidemiologic Research (RGE) requirements and are marked with an asterisk. Cancer incidence calculated in one hundred and seventy-six first-degree relatives

Second-degree relatives of G allele carriers had an overall significantly increased risk for developing cancer (RR = 1.50, *p* = 0.007, CI 1.15–2.01), and specifically for developing colorectal cancer (RR = 2.21, *p* = 0.043, CI 1.04–4.55) (Table 4).

**Table 4:** Cancer incidence for 2nd degree relatives of G allele carriers

Cancer Type	Obs	Exp	1 T P-Value	RR	95% CI
ANY CANCER	44	29.4	0.007	1.5	1.15–2.01
BRAIN	≤5	*	0.603	0	0–5.92
- OLIGODENDROGLIOMA	≤5	*	0.972	0	0–106.05
COLORECTAL	7	3.17	0.043	2.21	1.04–4.55
THYROID	≤5	*	0.114	3.48	0.62–12.56
PROSTATE	7	6.2	0.426	1.13	0.53–2.33

*Abbreviations:* *Obs* observed number of individuals with given cancer, *Exp* expected number of individuals with given cancer, *1 T P-Value* one-tailed *p*-value; *RR* relative risk, *CI* confidence interval

For all cancer sites with ≤5 observed cases, exact values are not shown for observed and expected to preserve patient anonymity per the Utah Resource for Genetic and Epidemiologic Research (RGE) requirements and are marked with an asterisk. Cancer incidence calculated in four hundred and twelve second-degree relatives

Again, no increased cancer risk was seen in second-degree relatives of rs55705857 A allele homozygote individuals (Table 5).

**Table 5:** Cancer incidence for 2nd degree relatives of rs55705857 A allele homozygote individuals

Cancer Type	Obs	Exp	1 T P-Value	RR	95% CI
ANY CANCER	43	34.73	0.097	1.24	0.94–1.67
BRAIN	≤5	*	0.569	0	0–5.31
- OLIGODENDROGLIOMA	≤5	*	0.970	0	0–97.08
COLORECTAL	≤5	*	0.338	1.31	0.51–3.05
THYROID	≤5	*	0.554	0	0–5.07
PROSTATE	8	7.87	0.529	1.02	0.51–2.00

*Abbreviations:* *Obs* observed number of individuals with given cancer, *Exp* expected number of individuals with given cancer, *1 T P-Value* one-tailed *p*-value, *RR* relative risk, *CI* confidence interval

For all cancer sites with ≤5 observed cases, exact values are not shown for observed and expected to preserve patient anonymity per the Utah Resource for Genetic and Epidemiologic Research (RGE) requirements and are marked with an asterisk. Cancer incidence calculated in four hundred and thirty-six second-degree relatives

## Discussion

We report a novel association between the rs55705857 G allele and multiple cancers. It is possible that this risk allele is not responsible for these cancers and that instead cosegregation between the rs55705857 G allele and one or more risk-associated single-nucleotide-polymorphisms in 8q24 is responsible for the excess prostate and colon cancer in our patient’s families [26–30]. However, studying a population of individuals with rare cancers and extended family history data may have allowed us to detect an association missed by previous GWAS and whole genome-sequencing studies [26–30]. Given that screening recommendations for prostate and colon cancer in high risk populations already exist, confirmation of this possibility in larger studies is warranted.

The ability to use tumor DNA for germline genotypes greatly expands the population available for familiality studies involving the 8q24 region. With tumor DNA, there is always a concern that somatic mutations or deletions will mask accurate genotypes and that tumor genotype may change over time [3, 31]. The confidence intervals on our results suggest that that the accuracy of tumor DNA from initial surgeries for genotype analysis of the rs55705857 G allele in tumor is at least ~ 80%. Our result of high accuracy in 8q24 tumor DNA is in line with previous literature indicating that this region may contain gliomagenesis driver mutations that are preserved and rarely deleted in gliomas [5]. Our results cannot assess whether tumor genomic evolution would affect this accuracy in tumor samples from reresection after chemotherapy and/or radiation.

Limitations of this study include the rarity of glioma in the general population and small number of tissue samples available in the CCR, which leads to large confidence intervals. Data censoring is present due to 61 samples failing to link to genealogy data and lack of data on cancers treated outside of Utah or diagnosed before 1966. However, from previous studies the number of such cancers is expected to be low [25]. Selection bias due to unknown confounders may be present but clinical factors such as age were not significantly different between individuals linked to genealogy data and those unlinked. Confirmation of our findings in other independent datasets is needed to validate our findings and refine risk estimates.

Although we expect 50% of first-degree relatives of G allele carriers to carry the G allele, first and second-degree relatives were not genotyped, which might have diluted the results for association between a high-risk allele and cancer incidence. It is possible that the risk of cancer in people who carry the G allele and who have a first degree relative with an *IDH* mutated glioma is twice as high as estimated. Greater sample size is needed to confirm these preliminary results.

## Conclusion

This was the first epidemiological study estimating cancer risks among first and second-degree relatives of rs55705857 G allele carriers [32]. Our population-based analysis confirms and extends previously published results associating the rs55705857 G allele with *IDH* mutated gliomas [5, 13, 15, 32–34]. We provide the first evidence in a prospectively identified cohort of this association.

We showed for the first time that relatives of rs55705857 G allele carriers have an increased risk of any cancer and especially of oligodendrogliomas, while relatives of A allele homozygote carriers had no increased risk of any cancer. A link between the G allele of rs55705857 and both prostate and colon cancer was suggested, but these results should be confirmed in an independent population [26, 27]. This manuscript provides evidence that allele status at rs55705857 is stable in tumors, confirms an increased risk of oligodendroglioma in first-degree relatives of rs55705857 G allele carriers, and suggests that further study of the role of the G allele in other cancers is warranted. Future work identifying the cancer risk based on relatives’ genotype and on identifying the *IDH* status in non-gliomas associated with the rs55705857 G allele is warranted.

## Additional file

Additional file 1: **Table S1:** DNA sample characteristics of glioma patients harboring *IDH1/2* mutant or 1p/19q co-deletion. **Legend:** Shown are the genotype results of 102 unique individuals. Samples were submitted in 96-well plates. Fifteen water blanks and replicate samples were plated at random. Cells highlighted in blue were called G Allele positive for rs55705857. Cells highlighted in orange have discrepancy between G allele status at rs55705857 in blood and tumor samples. Blood is germline and thus was held to be the true representation of an individual’s G allele status at rs55705857. (PDF 315 kb)

## Abbreviations

1 T *P*-Value: one-tailed *p*-value
CCR: Huntsman cancer institute cancer clinical research database
CI: confidence interval confidence interval
Exp: expected number of individuals with given cancer
HCI: Huntsman cancer institute
IDH: Isocitrate dehydrogenase family of genes
IRB: Institutional review board
Obs: observed number of individuals with given cancer
RGE: The Utah resource for genetic and epidemiologic research
RR: relative risk
SEER: Surveillance, epidemiology and end results
UCR: Utah cancer registry
UPDB: The Utah population database
