# Supplementary material for: The contribution of the rs55705857 G allele to familial cancer risk as estimated in the Utah population database
Source: BMC Cancer. 2019 Mar 1;19:190. doi: 10.1186/s12885-019-5381-2 (PMC6397494; doi:10.1186/s12885-019-5381-2)
Supplement: Supplementary file 1 — Table S1: DNA sample characteristics of glioma patients harboring IDH1/2 mutant or 1p/19q co-deletion. Legend: Shown are the genotype results of 102 unique individuals. Samples were submitted in 96-well plates. Fifteen water blanks and replicate samples were plated at random. Cells highlighted in blue were called G Allele positive for rs55705857. Cells highlighted in orange have discrepancy between G allele status at rs55705857 in blood and tumor samples. Blood is germline and thus was held to be the true representation of an individual’s G allele status at rs55705857. (PDF 315 kb) [file 12885_2019_5381_MOESM1_ESM.pdf]

Additional Table 1: DNA sample characteristics of glioma patients harboring *IDH1/2* mutant or 1p/19q co-deletion

Qubit ng/uL	Concentration	Source	Replicated	rs55705857 genotype	147958197 genotype	rs55705857 genotype run 2
94.3	determined at University of Utah	tumor	no	Likely AG	AG	AG
112	determined at Mayo	blood	replicate	AA	AA	AA
112	determined at Mayo	blood	yes	AA	AA	AA
35.6	determined at University of Utah	tumor	no	AA	AA Likely	AA
248	determined at University of Utah	tumor	no	equivocal	AA Likely	Failed
10.9	determined at University of Utah	tumor	no	equivocal	Failed	AA
317	determined at Mayo	tumor	no	AA	AA	AA
150	determined at Mayo	blood	no	AG	AG	AG
84.7	determined at Mayo	tumor	no	AG	AG	AG
46.1	determined at University of Utah	tumor	yes	AA	Failed	AA
46.1	determined at University of Utah	tumor	replicate	AA	Failed	AA
28.2	determined at University of Utah	tumor	no	AG	Failed	het gain of A
68.8	determined at University of Utah	tumor	no	AA	AA Likely	AA
51.6	determined at University of Utah	tumor	no	equivocal	Failed	Failed
99.7	determined at Mayo	blood	no	AA	AA	AA
132	determined at University of Utah	tumor	no	equivocal	Failed	Likely AA
494	determined at University of Utah	tumor	no	AG	AA	AG
192	determined at University of Utah	tumor	no	AA	AA Likely	AA
276	determined at Mayo	tumor	no	GG	AG	GG
108	determined at Mayo	blood	no	Failed	AA	equivocal
560	determined at University of Utah	tumor	no	AA	AA Likely	AA
469	determined at University of Utah	tumor	no	GG	het gain of G	Likely GG

Additional Table 1 Continued:

Qubit ng/uL	Concentration	Source	Replicated	rs55705857 genotype	147958197 genotype	rs55705857 genotype run 2
140	determined at Mayo	blood	no	AA	AA	AA
43	determined at University of Utah	tumor	no	AA	Failed	AA
159	determined at Mayo	blood	no	AA	AA	AA
188	determined at Mayo	tumor	no	AA	AA	AA
108	determined at Mayo	blood	no	AG	AG	AG
366	determined at University of Utah	tumor	no	AG	AG Likely	Likely AG
116	determined at Mayo	blood	no	equivocal	AG	AG
146	determined at Mayo	tumor	no	Likely AG	AG	AG
126	determined at Mayo	blood	no	AA	AA	AA
59.5	determined at University of Utah	tumor	no	equivocal	Failed	AA
too low	determined at Mayo	blood	no	AA	AA	AA
151	determined at Mayo	tumor	no	AA	AA	AA
116	determined at Mayo	blood	no	AA	AA	AA
165	determined at Mayo	tumor	no	AA	AA	AA
42.7	determined at University of Utah	tumor	no	AA	Failed	AA
110	determined at Mayo	blood	no	AA	AA	AA
350	determined at Mayo	tumor	no	AA	AA	AA
388	determined at Mayo	tumor	no	AG	AA	AG
15.4	determined at University of Utah	tumor	no	AA	Failed	AA
24.8	determined at University of Utah	tumor	no	AG	AG Likely	het gain of A
102	determined at University of Utah	tumor	no	AA	Failed	AA
110	determined at Mayo	blood	no	AG	AG	AG
115	determined at University of Utah	tumor	no	AG	AG Likely	AG
890	determined at University of Utah	tumor	no	AA	AA	AA

Additional Table 1 Continued:

Qubit ng/uL	Concentration	Source	Replicated	rs55705857 genotype	147958197 genotype	rs55705857 genotype run 2
87.9	determined at Mayo	blood	no	AG	AG Likely	AG
89	determined at Mayo	blood	no	Likely AA	AA	AA
103	determined at Mayo	blood	no	AG	AA	AG
37	determined at University of Utah	tumor	no	AG	Failed	AG
46	determined at University of Utah	tumor	no	AA	AA Likely	AA
37.3	determined at University of Utah	tumor	replicate	AA	AA Likely	AA
37.3	determined at University of Utah	tumor	yes	AA	AA Likely	AA
108	determined at Mayo	blood	no	AG	AG	AG
257	determined at University of Utah	tumor	no	AG	het gain of A	het gain of A
105	determined at Mayo	blood	no	AG	AG	AG
143	determined at University of Utah	tumor	no	AG	AG Likely	AG
106	determined at University of Utah	tumor	no	Failed	Failed	Likely GG
17.63	determined at University of Utah	tumor	no	equivocal	Failed	AA
119	determined at Mayo	blood	replicate	AA	AA	AA
119	determined at Mayo	blood	yes	AA	AA	AA
230	determined at Mayo	tumor	no	AA	Failed	AA
42.1	determined at University of Utah	tumor	no	AA	AA Likely	AA
77.9	determined at University of Utah	tumor	no	AA	AA Likely	AA

Additional Table 1 Continued:

Qubit ng/uL	Concentration	Source	Replicated	rs55705857 genotype	147958197 genotype	rs55705857 genotype run 2
86.8	determined at Mayo	blood	no	AA	AA	AA
102	determined at Mayo	tumor	no	AA	AA	AA
214	determined at University of Utah	tumor	no	GG	Failed	Likely GG
112	determined at Mayo	blood	no	AA	AA	AA
29.8	determined at University of Utah	tumor	no	equivocal	Failed	Likely AA
94.2	determined at Mayo	blood	yes	AG	AG	AG
94.2	determined at Mayo	blood	replicate	Likely AG	AG	Likely AG
33.5	determined at University of Utah	tumor	no	Likely AG	Failed	het gain of A
126	determined at Mayo	blood	no	AG	AG	AG
74.3	determined at University of Utah	tumor	no	Likely AG	AG Likely	AG
46.8	determined at University of Utah	tumor	no	AG	Failed	Likely AG
40.3	determined at University of Utah	tumor	no	AA	Failed	AA
141	determined at Mayo	blood	no	AA	AA	AA
71.6	determined at University of Utah	tumor	no	AA	AA Likely	AA
121	determined at Mayo	blood	replicate	Likely AA	AA	AA
121	determined at Mayo	blood	yes	AA	AA	AA
683	determined at Mayo	blood	yes	AA	AA	AA
133	determined at Mayo	blood	no	AA	AA	AA
683	determined at Mayo	blood	replicate	AA	AA	AA
32.4	determined at Mayo	blood	no	AG	AG	AG
32.1	determined at Mayo	tumor	no	Likely AA	AA	AA
234	determined at University of Utah	tumor	no	AA	Failed	AA
31.4	determined at University of Utah	tumor	yes	AA	AA Likely	Likely AA
31.4	determined at University of Utah	tumor	replicate	equivocal	AA Likely	AA
19.4	determined at University of Utah	tumor	replicate	AG	Failed	het gain of A
19.4	determined at University of Utah	tumor	yes	AG	Failed	het gain of A
96.4	determined at University of Utah	tumor	no	Failed	AA Likely	Likely AG

Additional Table 1 Continued:

Qubit ng/uL	Concentration	Source	Replicated	rs55705857 genotype	147958197 genotype	rs55705857 genotype run 2
117	determined at Mayo	blood	no	AG	AG	AG
47.6	determined at University of Utah	tumor	no	AG	Failed	het gain of A
143	determined at Mayo	blood	no	Failed	Failed	AA
353	determined at Mayo	tumor	yes	AA	AA	AA
353	determined at Mayo	tumor	replicate	AA	AA	AA
67.8	determined at University of Utah	tumor	no	AG	AG Likely	Likely AG
123	determined at Mayo	blood	no	equivocal	AA Likely	AA
112	determined at Mayo	blood	no	AA	AA	AA
159	determined at Mayo	blood	no	AA	Failed	AA
166	determined at Mayo	tumor	no	AA	AA	AA
too low	determined at Mayo	blood	no	AA	AA	AA
223	determined at Mayo	tumor	no	AA	AA	AA
75.8	determined at Mayo	blood	no	AA	AA	Likely AA
235	determined at Mayo	tumor	no	GG	het gain of G	GG
110	determined at Mayo	blood	no	AA	AA	AA
139	determined at Mayo	blood	no	AG	AG	AG
99.6	determined at Mayo	tumor	no	Likely AG	AG	AG
153	determined at Mayo	blood	no	AG	AA	AG
152	determined at Mayo	tumor	no	AG	AA	het gain of G
131	determined at Mayo	blood	no	equivocal	AA	AA
301	determined at Mayo	tumor	replicate	AA	AA	AA
301	determined at Mayo	tumor	yes	AA	AA	AA

Additional Table 1 Continued:

Qubit ng/uL	Concentration	Source	Replicated	rs55705857 genotype	147958197 genotype	rs55705857 genotype run 2
240	determined at Mayo	blood	no	AA	AA	Likely AA
378	determined at Mayo	tumor	no	AA	AA	AA
97.6	determined at Mayo	blood	no	AG	AG	AG
122	determined at Mayo	blood	no	Likely AG	AA	AA
412	determined at Mayo	tumor	no	AA	AA	AA
163	determined at Mayo	blood	no	AA	AA	AA
118	determined at Mayo	tumor	no	Failed	AA	AA
217	determined at University of Utah	tumor	no	AA	AA Likely	Likely AA
53	determined at Mayo	blood	no	AA	AA	AA
279	determined at Mayo	tumor	no	AA	AA	AA
103	determined at Mayo	tumor	no	AG	AA	AG
221	determined at University of Utah	tumor	no	equivocal	AG	Likely AG
166	determined at Mayo	blood	no	AA	AA	AA
295	determined at University of Utah	tumor	no	AA	Failed	AA
43.3	determined at University of Utah	tumor	replicate	GG	het gain of A	GG
43.3	determined at University of Utah	tumor	yes	Failed	het gain of A	Likely GG
153	determined at Mayo	tumor	no	AG	AA	Likely AG
60.5	determined at Mayo	blood	no	AG	Failed	AG
190	determined at Mayo	tumor	no	AA	AA	AA
38.2	determined at Mayo	blood	replicate	Failed	AG	AG
38.2	determined at Mayo	blood	yes	AG	AG	AG
277	determined at Mayo	tumor	no	AG	AG	AG

Supplementary Table 2 Continued:

Qubit ng/uL	Concentration	Source	Replicated	rs55705857 genotype	147958197 genotype	rs55705857 genotype run 2
12.2	determined at Mayo	blood	no	AA	AA	AA
73.3	determined at Mayo	tumor	no	AA	AA Likely	AA
108	determined at Mayo	blood	no	Likely AA	AA	AA
157	determined at Mayo	blood	no	AA	AA	AA
217	determined at Mayo	tumor	no	AA	AA	AA
186	determined at Mayo	tumor	no	AA	AA	AA
111	determined at Mayo	blood	no	AA	AA	AA
130	determined at Mayo	blood	no	AG	AA	equivocal
18.7	determined at Mayo	blood	no	GG	AG	GG
119	determined at Mayo	blood	no	AG	AG	AG
118	determined at Mayo	blood	no	AA	AA	AA
166	determined at Mayo	tumor	no	AA	AA	Failed
233	determined at Mayo	blood	no	AA	AA	AA
92.9	determined at Mayo	tumor	yes	Likely AA	AA	AA
92.9	determined at Mayo	tumor	replicate	AA	AA	AA
118	determined at Mayo	blood	no	AG	AG	AG
115	determined at Mayo	blood	no	AG	AG	AG
134	determined at Mayo	blood	replicate	AG	AG	Likely AG
134	determined at Mayo	blood	yes	AG	AG	AG
107	determined at Mayo	blood	no	AA	AA	AA
124	determined at Mayo	tumor	no	AA	AA	AA
water	water	water	water	Failed	Failed	Failed
water	water	water	water	Failed	Failed	Failed
water	water	water	water	Failed	Failed	Failed
water	water	water	water	Failed	Failed	Failed

Shown are the genotype results of 102 unique individuals. Samples were submitted in 96-well plates. Fifteen water blanks and replicate samples were plated at random. Cells highlighted in blue were called G Allele positive for rs55705857. Cells highlighted in orange have discrepancy between G allele status at rs55705857 in blood and tumor samples. Blood is germline and thus was held to be the true representation of an individual's G allele status at rs55705857.
